# Perspectives on the Training of Chinese Primary Health Care Physicians to Reduce Chronic Illnesses and Their Burden

**DOI:** 10.3389/fpubh.2019.00168

**Published:** 2019-07-02

**Authors:** Wenmin Sun, Yang Li, Yiting Hu, Xin Rao, Xingzhi Xu, Colette Joy Browning, Shane Andrew Thomas

**Affiliations:** ^1^Health and Family Planning Capacity Building and Continuing Education Center of Shenzhen Municipality, Shenzhen, China; ^2^Shenzhen International Primary Health Care Research Institute, Shenzhen, China; ^3^School of Nursing and Healthcare Professions, Federation University, Ballarat, VIC, Australia; ^4^Research School in Population Health, Australian National University, Canberra, ACT, Australia

**Keywords:** Chinese primary health care physicians, training, chronic illness, China, burden of disease

## Abstract

This paper is a commentary on the training of Chinese Primary Health Care Doctors to reduce chronic illness and its burden. First, we will consider the policy position of the Chinese government concerning the development of a competent and enlarged primary physician workforce to deliver the proposed primary health care system reforms. We then turn to a review of the drivers of the high burden of chronic illnesses especially in older people in China. We argue that the curriculum for the training of primary health care medical practitioners should match the demonstrated high prevalence chronic illnesses and their risk factors and that there needs to specific competencies in prevention and mitigation of the diseases and their risk factors.

## Policy context of China's Primary Health Care Reforms

**Figure 1:**
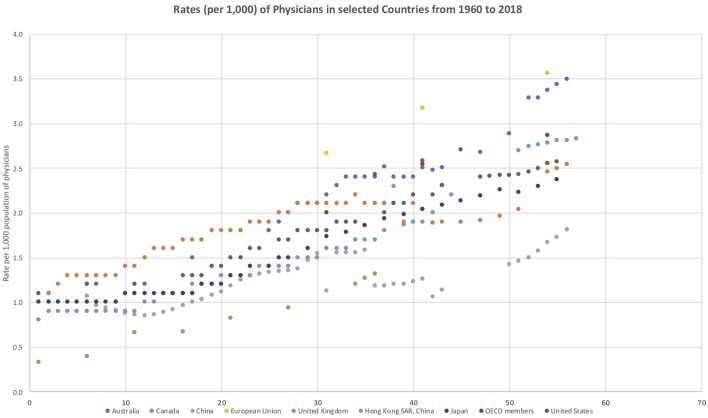
Rates (per 1,000) of Physicians in selected Countries from 1960 to 2018.

The various policy statements from the Chinese State Council (1) and the responsible ministries (2) regarding the physician workforce in China are clear and consistent. China needs more qualified doctors, especially general practitioners who have the skills required to meet the needs of the people and their population health needs. World Bank Indicators data (3) show that while China has experienced rapid growth in the size of its physician workforce, the OECD economies currently have approximately 150 to 200 per cent of the per capita (per 1,000 population) physician workforce compared to China and many OECD countries also consider themselves to be in a position of doctor shortage. The following figure shows the historical trends of physician ratios per 1,000 people from 1960 to 2018 for China and other key countries involved in primary health care reform (Figure 1).

China has also recognized the need for expansion of its primary care physician workforce and has set ambitious targets for the growth in this workforce (4). Its physician workforce is currently predominantly composed of specialists and there is a desire to modify this situation to increase the primary care physician workforce proportion.

## The Health Needs and Burden of Disease Within the Chinese Population

In designing training systems for the Chinese primary care physician workforce it is necessary to understand the health needs of the people that the workforce is intended to service (5). The population health needs of the Chinese people are very well-documented in a many academic and government reports. Chronic illness in China is a major burden as it is in most countries globally. Sufficient numbers of well-trained doctors are needed to deal with what the World Health Organization (6) has labeled a “tsunami” of chronic illnesses currently being experienced in China and globally (7, 8). It is widely agreed that the Chinese health system needs to be able to prevent and reduce the rates and numbers of new cases of chronic illness and Non-Communicable Diseases and to mitigate the severity of existing cases of chronic and Non-Communicable Diseases.

The United Nation's Sustainable Development Goals for 2030 (9) include reducing premature mortality from non-communicable diseases (NCDs) by one third for all countries including China. This is an ambitious target. Li et al. (10) study specifically considers the feasibility of achievement of the United Nations Sustainable Development 30 percent reduction goal for China. The researchers used the 2013 Global Burden of Disease Study data from China to model projected premature mortality in 2030 of NCDs using different risk factor reduction scenarios. The risk factors used were high systolic blood pressure, smoking, high body mass index (BMI), high total cholesterol, physical inactivity, and high fasting glucose. The researchers found that if the current trends for each of these risk factors in China continued to 2030, then total premature deaths from NCDs would increase from 3.11 million to 3.52 million people. However, if the risk reduction targets were reached then one million deaths among persons 30 to 70 years old in China due to NCDs would be avoided. The authors argue that more strenuous efforts are required to achieve risk factor reduction. This is a widely shared view.

## The Deepening Reform Study and its Implications

The China Joint Study Partnership comprising the World Bank Group, the World Health Organization, the China Ministry of Finance, the National Health and Family Planning Commission, the Ministry of Human Resources and Social Security (2) recently released a comprehensive analysis of Chinese Health Service Reform in its Deepening Reform study program. The Deepening Reform analysis confirms the same issues raised in State Council policy directives and the large body of academic research that has focused on the health of the Chinese population. The report notes the important impact of the rapidly aging profile of China upon burden of disease in China and that China will soon overtake many OECD countries in its population proportion of older people. The report notes the rapid growth in the impact of NCDs and chronic illness upon the health of the Chinese population. It is noted that NCDs are responsible for 77 percent of the loss in healthy life and 85 percent of all deaths and that this trend is increasing. The report also comprehensively analyses the impact of risk factors upon the growth in chronic disease burden. It notes that, while population aging is important that, across the whole Chinese population, key high-risk behaviors including smoking, poor nutrition, low physical activity, hazardous alcohol consumption, and environmental factors are the key drivers of increasing disease burden for the whole population. The analyses identify the same issues in many of the other analyses of the epidemiology of the Chinese population.

However, the Deepening Reform report (2) differs from many others in that it also includes a deep analysis of potential policy measures and actions that may be taken to improve China's situation. The report identifies a set of influential “levers” for reform. These are:

1. Shaping a tiered health care delivery system in accordance with People-Centered Integrated Care models
2. Improving quality of care in support to People-Centered Integrated Care
3. Engaging citizens in support of People-Centered Integrated Care
4. Reforming public hospitals and improving their performance
5. Realigning incentives in purchasing and provider payment
6. Strengthening health work force for People-Centered Integrated Care
7. Strengthening private sector engagement in production and delivery of health services
8. Modernizing health service planning to guide investment.

The central inclusion of the concept of People-Centered Integrated Care reflects best global health care practice (11). This is the way that many health systems have gone, by placing the patient at the center of the health service efforts and design. This approach has delivered substantial benefits in program effectiveness and efficiency (see, for example, the Cochrane Collaboration Systematic review database https://www.cochranelibrary.com/ which at the time of writing had 162 systematic reviews of patient centered care with almost universally successful results). A further key feature of the Deepening Reform report is its evidence-based philosophy. The report focusses on what can be done to improve the efforts in the delivery of services that address the demonstrated health needs of the Chinese people.

## Training Considerations for China's Primary Health Care Medical Workforce

While all the identified “levers” are certainly worthy of attention, our focus as educators of primary health care physicians in this commentary is on delivering the required numbers of appropriately trained doctors to participate in the new reformed Chinese system. This raises the question of what should be included in an appropriate Chinese primary care physician curriculum?

We have made substantial progress in the development of the Shenzhen standards for primary care physicians. We have critically analyzed the major Western General Practice clinical standards. The new Shenzhen standard was developed using rigorous modified Delphi procedures with a large expert panel building on a detailed analysis of relevant international standards. Embedded in the standards are assumptions about what doctors will and should do in their everyday practice. We have separately reported the Shenzhen standards development in another publication^1^ but a key feature of our strategy has been matching the standards content to meet the health needs of the Chinese population as well as matching or exceeding contemporary international standards. It is obvious that the prevention and management of chronic illness and non-communicable diseases must be a key focus in a primary care physician's training curriculum. There are two aspects to this focus. The first aspect is content knowledge about the most prevalent and burdensome conditions that afflict the community. The content needs to be evidence-based using the best epidemiological data concerning disease burden and also it needs to be evidence based in terms of the selection of effective treatments to treat these diseases.

The major illnesses associated with China's disease burden have been studied in detail by many government bodies and academic researchers. The same lists emerge from these analyses. The Deepening Reform Study (2) and the World Health Organization (12) China country assessment report on aging and health have considerable commonality as they draw from the same authoritative data sets. The analyses presented in the WHO country assessment (12) estimate that the following conditions are associated with the highest disease burden. Burden of disease is typically measured by Disability Adjusted Life Years (13) and the WHO country assessment (12) provides the following estimates for various conditions for older people:

1. Stroke (35.9 million DALYs)
2. Malignant neoplasms (30 million DALYs);
3. Ischaemic heart disease (22.6 million DALYs);
4. Respiratory diseases (16 million DALYs);
5. Diabetes mellitus (5.6 million DALYs);
6. Mental health conditions such as depression, suicide and dementia (5.3 million DALYs);
7. Hypertensive heart diseases (3.6 million DALYs); and
8. Falls (3 million DALYs).

Thus, it is evident that primary health care physicians require a substantive knowledge of these conditions and their prevention and management. This is how many medical curriculums are organized with a list of key conditions to be covered.

## A Focus on Risk Factors and Their Mitigation

However, we consider that the second key focus is the need for primary care physicians to identify and to mitigate and control the risk factors that exacerbate the main diseases. To mitigate these risk factors requires a quite specific skill set. The same analyses have identified risk behaviors amongst the Chinese population that exacerbate the above list of diseases. For example, smoking is a key health risk for most of the above list of diseases/ conditions. But there are others that the WHO analysis (8) has identified. These include:

1. Dietary risks
2. High blood pressure
3. Smoking
4. High fasting glucose levels
5. Exposure to air pollution and
6. Physical inactivity

While our focus for this paper is behavioral risk factors that can be mitigated in the primary health care setting, in order to address behavioral risk factor mitigation in China we recognize that multi-level approaches are needed including a systematic approach to chronic illness management within the health system, public health interventions, as well as primary health care physician training in behavior change approaches. In China, as discussed previously, the Deepening Health Reform in China monograph (2), developed by the World Bank, the WHO and various senior Chinese ministries and commissions, highlights a number of proposed levers to improve health outcomes in China, including a Patient-Centered Integrated Care Model, and engaging patients in their care, which build on the experience of Western countries.

In the United Kingdom and Australia, chronic illness management systems have been strongly influenced by the Wagner Chronic Care Model (14) and a focus on patient centered care (15). These approaches include an emphasis on team-based care and better coordination (integration) across primary care settings and hospitals, integrated patient record systems and patient access to their health records, medication management and self-management support.

In Australia, General Practitioners (GPs) are funded by the national health insurance system and government to provide longer consultations and care planning for patients with chronic illnesses (16). A recent review of confirms that Self-Management Support is the most frequent Chronic Care Model intervention that is associated with statistically significant improvements, predominately for diabetes and hypertension (17). Self-Management support requires the ability of the primary care physician to manage behavior change in their patients. This has been long recognized in some countries. For example, the Royal Australian College of General Practitioners have for 20 years developed and revised standards and support resource materials to assist general practice doctors to assist their patients to modify these risk factors through behavior change principles (18). The United Kingdom has also strongly promoted behavior change in its approaches to chronic disease risk reduction (19).

Medication management in primary care in people with chronic illnesses has been a particular focus in Australia and world-wide. Polypharmacy, the prescription of potentially inappropriate conflicting medications has been recognized a potential risk to patients with chronic illness and the typical pattern of multimorbidity. However, the effectiveness of polypharmacy reduction programs (20–22) and the process of de-prescribing (23) has been shown to have quite varied research outcomes, notwithstanding the widespread acceptance of the necessity to reduce it. Polypharmacy in China is a significant problem particularly in older patients (24). Specific issues in China driving polypharmacy include the dominant role of the doctor in the doctor-patient interaction and low patient health literacy, the profit-driven nature of medical interventions in China, and stong pharmaceutical marketing (25). The National Essential Medicine System has shown some promise in improving the rational use of medicines in primary care settings in China (26).

Chronic illness management also needs to address mental health co-morbidities. Since 2006 the Chinese Government policy has promoted the detection and treatment of mental illness in primary care settings through integrated mental services (27). Yet training in mental health for primary care physicians in China is insufficient (28) and patient perceptions including stigma and concerns about the competency of primary care physicians are barriers to treatment (29). Li et al. (30) reviewed the prevalence of co-morbid depression in people with chronic illnesses (COPD, diabetes, stroke, heart disease and cancer) and treatment rates in the US and China. Depression prevalence rates ranged for 40 to 80% across the diseases. Depression prevalence rates were higher in China for diabetes, heart disease and cancer and higher in the US for COPD and stroke. Based on 2009 data, in the US 47% of people with depression were detected by primary care physicians and 35% received treatment. Based on 2003 data treatment levels in China are significantly lower with only 1% of people with depression being treated. While Li et al. (29) focused on the implications of these findings for nurses their general conclusion was that primary care providers needed further skills development in the areas of detecting and managing mental health issues in the context of chronic illness management.

Public health approaches are also an important part of addressing behavioral risk factors at the population level. Smoking is an example of the need for combined regulatory, public health and primary care approaches. For example, to reduce smoking rates, strategies such as increasing excise on tobacco, regulating smoking advertising, banning smoking in public places, public health education and health promotion programs and plain paper packaging have been used in Australia and other western countries to reduce smoking levels (31). In China, these approaches have had limited impact on smoking rates. A Quitline model was introduced in China in 2004 and provided information and counseling calls. However, the effectiveness of the approach was limited by low funding and a lack of targeted approaches for specific at-risk populations (32). Smoke-free legislation in public places was introduced in Guangzhou, China. Where full smoking bans were introduced self-reported smoking decreased in younger age groups (33). A recent analysis argued that in addition to non-price measures, to reduce smoking targets under Healthy China 2030 (34) tobacco excise would need to be doubled (35). However, while public health and more general public policy approaches have been successful in countries such as Australia, these have been complemented and supported by more individual level clinical care approaches provided within the primary health care setting, an approach that could benefit smoking reduction in China.

Addressing physical inactivity is another important risk factor for poor health and chronic illness prevention and management that requires primary care and public health interventions as well as legislative approaches. In a review of national policies to increase Health Enhancing Physical Activity (HEPA) in seven European countries, Bull et al. (36) reported a number of findings relevant to public health and legislative approaches to inactivity. Several countries had specific physical activity policies and all reviewed countries had legislation about mandatory physical education in schools. Some countries had legislation on transport and the environment relevant to HEPA. Six of the countries had national HEPA guidelines and targets, 5 had surveillance systems, and most used mass media campaigns. Evaluation of the HEPA programs was generally poor with little evidence of implementation processes. The authors concluded there was insufficient policy progress in this area. The Lancet 2016 series (37) on physical activity argued that physical activity goals need to reflect and be consistent with social, environmental, and sustainable development goals and embrace a multi-sectoral, multidisciplinary public health approach.

The Chinese National Fitness Plan (2016-2020) aims to increase physical activity and population health (38). The plan sets targets for physical activity (700 million participating in PA at least once a week, 435 million engaging in regular physical activity). However, Wu et al. (39) argue that in China consensus is lacking concerning the best forms of physical activity given availability and environmental issues associated with outdoor activities. Consistent with recommendations form the aforementioned Lancet series, the authors (38) recommended: a co-ordinated approach across public health authorities, health care, transportation and the environment, the development of mass campaigns, a research agenda to support the implementation of evidence-based programs and the establishment of national surveillance systems. Primary care physicians in China have a significant role to play in this co-ordinated approach similar to their counterparts in Western countries. Physical activity is a key component of primary care chronic disease supported self-management approaches. In order to fulfill this potential Chinese primary care physicians need training in behavior change approaches.

### The Evidence for the Success of Chronic Disease Risk Factor Mitigation in China

There has been significant Chinese work in clinical trials and systematic reviews of this issue. For example, we examined the therapeutic effects of motivational interviewing on blood pressure control by conducting a meta-analysis of randomized controlled trials (40). The review showed strong effects of the motivational interviewing technique in enabling blood pressure control in the RCTs studied. Similarly, members of our research group reviewed psychological interventions for the management of glycemic and psychological outcomes of Type 2 Diabetes Mellitus in China by conducting systematic review and meta-analyses of relevant Randomized Controlled Trials (41). We have also conducted our own RCTs of treatment effectiveness in the control of T2DM (42, 43) using these techniques and we have found them to be highly effective. There are many such studies that show strong positive effects of this type of intervention and this is why there is such interest in the use of these techniques in the clinical management of high risk behaviors.

## Conclusions

There is widespread agreement about the need to appropriately train general practitioner physicians so that they may deliver high quality and effective care to their patients. There is also strong evidence that there is a global shortage of skilled practitioners and China has particular needs to develop the talent pool to support its ambitious primary care reform agenda. We have argued that primary care physician training needs to be informed by rigorous analysis of the causes and underlying risk factors for high burden of disease conditions. These conditions and their treatments have been well-researched and there is a strong evidence base. We propose that to effectively prevent and mitigate the many chronic conditions and diseases that are caused and exacerbated by lifestyle factors requires high level behavior change skills, mental health detection and management skills and other patient management skills that need to be included in the Chinese primary care physician curriculum.

## Data Availability

Publicly available datasets were analyzed in this study. This data can be found here: http://datatopics.worldbank.org/world-development-indicators/

## Author Contributions

All authors listed have made a substantial, direct and intellectual contribution to the work, and approved it for publication.

### Conflict of Interest Statement

The authors declare that the research was conducted in the absence of any commercial or financial relationships that could be construed as a potential conflict of interest.

## Footnotes

^1^Rao X, Lai J, Wu H, Li Y, Xu X, Browning C, et al. (2019). A competency assessment standard and curriculum for General Practitioners in China. (under review).
